# Normal Anatomy and Anomalies of the Rectus Extraocular Muscles in Human: A Review of the Recent Data and Findings

**DOI:** 10.1155/2019/8909162

**Published:** 2019-12-28

**Authors:** Robert Haładaj

**Affiliations:** Department of Normal and Clinical Anatomy, Chair of Anatomy and Histology, Medical University of Lodz, Łódź, Poland

## Abstract

Development of modern surgical techniques is associated with the need for a thorough knowledge of surgical anatomy and, in the case of ophthalmologic surgery, also functional aspects of extraocular muscles. Thus, the leading idea of this review was to summarize the most recent findings regarding the normal anatomy and anomalies of the extraocular rectus muscles (ERMs). Particular attention was paid to the presentation of detailed and structured data on the gross anatomy of the ERMs, including their attachments, anatomical relationships, vascularization, and innervation. This issue of ERMs innervation was presented in detail, considering the research that has recently been carried out on human material using advanced anatomical techniques such as Sihler's technique of the nerves staining. The text was supplemented with a carefully selected graphic material (including anatomical specimens prepared specially for the purpose of this review) and discussion of the clinical cases and practical significance of the presented issues.

## 1. Introduction

The statement of Farzavandi [1], who concluded that “An intimate knowledge of anatomy, including the extraocular muscles, periocular fascia, and orbit, is necessary to be an accomplished strabismus surgeon,” became a motto of the presented review. Development of modern surgical techniques is associated with the need for a thorough knowledge of surgical anatomy and, in the case of ophthalmologic surgery, also functional aspects of extraocular muscles [1–6]. However, getting familiar with the numerous data that appear in various scientific journals may be difficult and time consuming. Hence the need and value of thorough literature reviews on specialized and clinically significant areas of the basic sciences.

The leading idea of this paper was to summarize the most recent findings regarding the normal anatomy and anomalies of the extraocular rectus muscles (ERMs) in human, that is, medial (MR), lateral (LR), superior (SR), and inferior (IR) rectus muscle. Particular attention was paid to the presentation of detailed and structured data on the gross anatomy of the ERMs, including their attachments, measurements, anatomical relationships, vascularization, and innervation. This issue of ERMs innervation was described in detail, including the recent research that has been carried out on human material using advanced anatomical techniques such as Sihler's technique of the whole mount nerve staining [2–6]. The text was supplemented with a carefully selected graphic material (including anatomical specimens prepared specially for the purpose of this review) and discussion of the clinical cases and practical significance of the presented issues.

## 2. Review of Anatomical Data Regarding ERMs

### 2.1. Attachments and General Arrangement of ERMs

**Figure 1:**
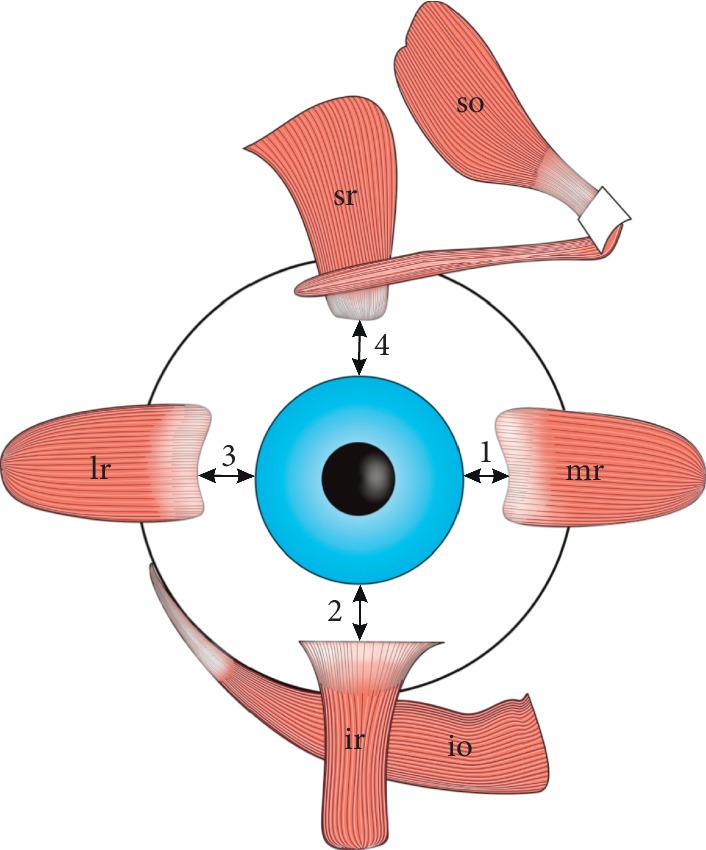
Arrangement of the extraocular rectus muscles (ERMs) around the equator of the eyeball. 1: the distance of the medial rectus muscle (mr) from the limbus; 2: the distance of the inferior rectus muscle (ir) from the limbus, 3: the distance of the lateral rectus muscle (lr) from the limbus; 4: the distance of the superior rectus muscle (sr) from the limbus. The results of measurements made by various authors on individual ERMs insertions slightly vary. However, it may be observed that the distance from the lateral rectus muscle insertion to the limbus is greater than the distance from the medial rectus muscle to the limbus, as well as the distance from the lateral rectus muscle insertion to the limbus is greater than the distance from the medial rectus muscle to the limbus. According to the classical data provided by Fuchs [14] the distances of the individual rectus muscles from the limbus increase counterclockwise starting from the medial rectus muscle.

The four ERMs have a specific attachment to the circular fibrous band located in the apex of the orbit, known as the common tendinous ring, annular tendon, or annulus of Zinn. All four ERMs take origin from this tendinous structure with short proximal tendons [7–14]. The common tendinous ring surrounds the optic nerve and some part of the superior orbital fissure (Figure 1). Therefore, the content of the ring is nerves and vessels running through above-mentioned spaces, that is, optic nerve and ophthalmic artery (which run through the optic canal), as well as superior and inferior branches (division) of the oculomotor nerve, the nasociliary nerve, and the abducens nerve (which run through the superior orbital fissure). Moreover, the annular tendon is also the place of attachment of the superior oblique muscle and levator palpebrae superioris muscle [7–14]. Zampieri et al. [12] indicate that the annular tendon was first described by Antonio Maria Valsalva in his treatise “Opera omnia” published in 1740. However, as indicated by Zampieri et al. [12], the first correct functional interpretation of the meaning of this structure was given by Johann Gottfried Zinn in “Descriptio anatomica oculi humani,” which was published in 1755.

On their further course, ERMs direct divergently towards the equator of the eyeball, with a cone-like arrangement around the optic nerve and posterior globe, forming the boundaries of the so-called intraconal space [15]. The length of ERMs is close to 40 mm, however, the tendons of those muscles differ in length: the MR is characterized by the shortest tendon (about 4 mm), while the tendon of the LR is the longest (about 8 mm) [1]. The average tendon lengths for the IR and SR are 6 mm and 7 mm, respectively. The distal tendons of all four ERMs occupy the anterior position regarding the eyeball's equator and are attached to the sclera [1, 7–14]. However, the insertions of the ERMs also vary in their specific position on the eyeball, their distance from the limbus, and the length of the distal tendon. The results of measurements made by various authors on individual ERMs insertions slightly vary (Figure 1) [1, 8–11, 13, 14]. Pihlblad et al. [13] measured distances between the ERMs insertions and the limbus in 46 patients who underwent anterior segment optical coherence tomography. According to results obtained by Pihlblad et al. [13] the mean distances between insertion and the limbus measured for each rectus muscle were as follows: 5.7 mm (SD = 0.8 mm, range from 4.3 mm to 7.8 mm) for the MR; 6.0 mm (SD = 0.6 mm, range from 4.8 to 7.0 mm) for the IR; 6.8 mm (SD = 0.7 mm; range from 4.8 mm to 8.4 mm) for the LR; and 6.8 mm (SD = 0.6 mm, range from 5.5 mm to 8.1 mm) for the superior rectus. Stärk and Kuck [14], in turn, performed their measurements during strabismus surgeries. Those authors found the following mean values: insertion-limbus distance of the MR = 4.5 mm (in 675 eyes operated on); insertion-limbus distance of the IR = 5.67 mm (in 22 eyes operated on); insertion-limbus distance of the LR = 6.20 mm (in 493 eyes operated on); and insertion-limbus distance of the SR = 6.64 (in 21 eyes operated on). Stärk and Kuck [14] compared their results with the classical data provided by Fuchs more than 100 years ago, who assessed the distances between insertion and limbus at 5.5 mm for the MR, 6.5 mm for the IR, 6.9 mm for the LR, and 7.7 mm for the SR. The general tendency observed by various authors [1, 8–11, 13, 14] seems to be that usually the distance between the insertion and limbus increases for subsequent ERMs when counting from the MR and moving counterclockwise (Figure 1). Farzavandi [1] gives one more indicator (i.e., “arc of contact”) that defines the relation of the distal tendon to the sclera. The arc of contact differs between specific ERMs and measures 6 mm for the MR, 7 mm for the IR, 10 mm for the LR, and 6.5 mm for the SR [1]. Summing up, the accurate visualization and location of ERMs' insertions may be crucial during ophthalmology surgeries [1, 13].

### 2.2. Basic Morphological Characteristics of Individual ERMs

#### 2.2.1. Medial Rectus Muscle

**Figure 2:**
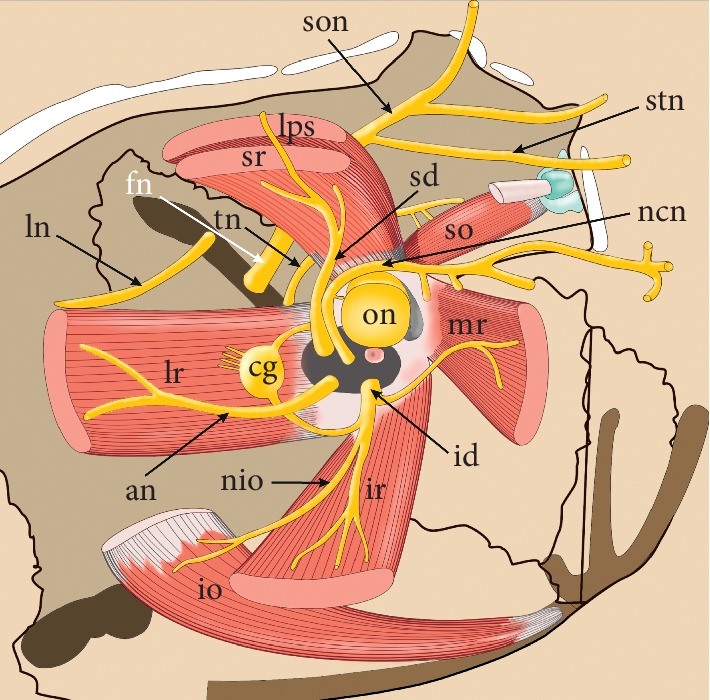
Anatomical relationships within the orbit. The origin of the medial rectus muscle (mr) is fixed to the medial aspect of the common tendinous ring. On its further course, the muscle runs parallel to the medial orbital wall. The inferior rectus muscle (ir) runs perpendicularly to the inferior wall of the orbit. The anterior part of the muscle is separated from the orbit by the inferior oblique muscle (io). All three muscles (mr, ir, and io) are innervated by the inferior division of the oculomotor nerve (id). The nerve to the inferior oblique muscle (nio) branches off from the inferior division of the oculomotor nerve and runs parallel to the lateral border of the inferior rectus muscle. A characteristic feature of the lateral rectus muscle (lr) is the so-called “dual headed origin,” as this muscle begins from the common tendon ring with two tendinous bands. The muscle runs along the lateral wall of the orbit. It is innervated by the abducens nerve (an). The ciliary ganglion (cg) is located medially to the internal surface of the muscle. The superior rectus muscle (sr) is located under the levator palpebrae superioris muscle (lps). Both muscles (sr and lps) are innervated by the superior division of the oculomotor nerve (sd). fn: frontal nerve; ln: lacrimal nerve; ncn: nasociliary nerve; son: supraorbital nerve; stn: supratrochlear nerve, th: troch; ear nerve.

The MR is the widest among all ERMs [2]. The muscle's origin is fixed to the medial aspect of the common tendinous ring. On its further course, the MR runs parallel to the medial orbital wall (Figure 2) and inserts to the sclera by tendinous expansion, which is about 10 mm wide at the level of insertion to the eyeball [1, 9]. The length of the MR tendon, measured from the origin, is about 3.7 mm, while the average entire length of the MR is about 40.8 mm [9]. Detailed measurements of the MR were performed by Shin et al. [5], who estimated the average entire length of the MR muscle belly at 37.6 mm (SD = 4.6 mm), MR tendon length at 4.4 mm (SD = 1.9 mm), and MR width at 10 mm (SD = 1.8 mm).

The MR acts as an adductor of the eye [1, 2, 9]. Together with the LR, the muscle is classified as so-called horizontal rectus muscles, due to its primary action (adduction of the eye) which occurs in the horizontal plane. Moreover, the horizontal vestibulo-ocular reflex requires coordinated activity of both the MR and LR [16].

#### 2.2.2. Lateral Rectus Muscle

A characteristic feature in the structure of the LR is the so-called “dual headed origin,” as this muscle begins from the annular tendon with two tendinous bands (Figure 2) [7]. The medial part of the superior orbital fissure is located between those two initial parts of the LR. The LR travels along the lateral wall of the orbit and inserts to the sclera by the tendinous expansion, which is 9.6 mm long on average [4, 6]. According to the latest research of Shin et al. [4], the mean length of the LR is 46 mm (SD = 4.5 mm), the average length of the LR muscular part is 36.4 mm (SD = 4.5 mm), while the average width of the muscle is 11.2 mm (SD = 1.6 mm). The line of insertion of the LR is vertical (sometimes slightly convex) and usually symmetrical [1].

Lateral movement of the eyeball is the primary function of the LR [1, 2, 8]. However, the recent studies indicate the complex activity of the LR, with the existence of the functional compartments (zones) within the muscle, which may be activated selectively depending on the type and phase of movement [2–4, 17–22].

#### 2.2.3. Superior Rectus Muscle

The SR is located under the levator palpebrae superioris muscle (Figure 2) and direct anteriorly and slightly laterally, to attach the sclera. The distance between the insertion of the SR and the limbus is about 7.7 mm which is the greatest out of all rectus muscles [8–11, 13, 14]. The muscle forms an angle of about 23 degrees with the visual axis. It acts during the elevation of the eye, as well as with adduction and medial rotation [10]. Along with the IR, the SR is classified as vertical rectus muscle.

The fibrous sheath covering the SR is thickened on its distal end, underlying the levator palpebrae superioris and fuses with the levator's sheath which is also thickened on its distal portion. The structure created in this way is referred to as conjoint fascial sheath [23, 24]. It extends forward between the two muscles; it has trapezoid shape and attaches to the superior conjunctival fornix [23]. According to Hwang [25], the average length of the conjoint fascial sheath is 12.2 mm (SD = 2 mm), while the average thickness of this structure is 1.1 mm (SD = 0.1 mm). Due to those close relations between the two muscles, the term “levator-superior rectus complex” is also used [26]. Thus, the proper function of the SR also affects the upper eyelid position [26, 27].

#### 2.2.4. Inferior Rectus Muscle

The IR runs perpendicularly to the inferior wall of the orbit (Figure 2). The long axis of the muscle also deviates from the visual axis (this line is tilted by approximately 23 degrees with the visual axis) [11]. The anterior part of the muscle is separated from the orbit by the inferior oblique muscle. The muscle causes depression (primary function), external rotation (secondary function), and adduction of the eyeball (tertiary function).

### 2.3. Anatomical Variations and Anomalies of ERMs

Individual ERMs have relatively constant morphology. Anatomical variability most often concerns the size (length, width, and thickness of muscle belly or tendon), as it was characterized in previous paragraphs [1, 7–11]. Individual ERMs may occasionally be duplicated, underdeveloped, or even absent. In addition, accessory muscular bands may occur inside the orbit [28]. However, anatomical variations and anomalies of ERMs are sporadically reported based on diagnostic imaging, as well as surgical or anatomical findings.

#### 2.3.1. Vertical Rectus Muscles

Gross anatomical variations of the SR are rarely observed. Nayak et al. [29] provided a case report on double-bellied SR found during routine dissection of a 70-year-old cadaver. Both parts of the SR had equal size, with separate origins from the common tendinous ring which united to form a common belly one centimeter before insertion of the muscle [29]. Bagheri et al. [30], in turn, reported unilateral SR aplasia in a patient clinically presenting an incomitant left hypotropia. The patient described by Bagheri et al. [30] showed no craniofacial anomalies. Also, Mather and Saunders [31] described a unique case of a patient with bilateral absence of SR, presenting clinically as a double elevator palsy. That case was unique due to the patient's “paradoxical ocular movements on attempted upgaze” and the absence of any major craniofacial anomalies [31]. According to Ingham et al. [32], the frequency of congenital extraocular muscle anomalies increases in craniofacial dysostosis. Also, Law et al. [33] stress that “reports of absent or anomalous SR muscles have occurred in the context of craniofacial diseases.” For instance, bilateral agenesis of the SR was reported in Apert's syndrome [34]. However, based on observations of Diamond et al. [35], the etiopathology of those anomalies and their frequency in craniofacial dysostosis are unknown.

Reports of absence of the IR are also available in the literature. However, it seems that such anomalies cause significant clinical manifestations. For example, Yang and Guo [36] described a series of five patients with hypertropia resulting from the absence of the IR. Astle et al. [37] suggest that congenital absence of the IR muscle may mimic IR palsy especially in the absence of associated craniofacial anomalies. A case of unilateral congenital absence of the inferior rectus muscle was described by Ingham et al. [32]. In a patient examined by Ingham et al. [32], an abnormality was diagnosed while planning vertical muscle surgery for a large left hypertropia. Except for the lack of the IR, rare reports of duplication of the muscle are known. Such case was presented by Shazly and Stefko [38], who described unilateral congenital entropion and hypotropia resulting from duplication of the IR.

Regarding normal conditions, anatomical relationships between SR and muscular branches to the levator palpebrae superioris muscle can also be variable. Occasionally, the SR may be pierced by some of those branches. Djordjevic et al. [39] reported that in 12.5% of cases the muscular branch to the levator palpebrae superioris may pass through the SR belly to innervate levator palpebrae superioris muscle. Bye et al. [40] also provides information that the SR may occasionally be pierced by nervous branch to the levator palpebrae superioris.

**Figure 3:**
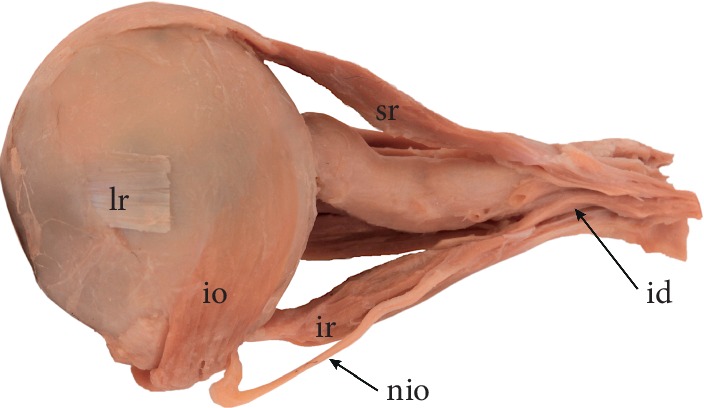
Specimen, in which the nerve to the inferior oblique muscle (nio) pierces the inferior rectus muscle (ir). Lateral view to the left eyeball. Lateral rectus muscle was removed, only the insertion of the lateral rectus (lr) is visible. When the nerve to the inferior oblique pierces the inferior rectus muscle, its susceptibility to injury associated with stretching of the muscle during surgical procedures seems to be greater. sr: superior rectus muscle; io: inferior oblique muscle.

Anatomical relations between the IR and the nerve to the inferior oblique muscle may also be variable. In some instances, the nerve to the inferior oblique muscle may pierce the IR (Figure 3). Black et al. [41] provide an interesting hypothesis about the clinical significance of such relationships. According to those authors, the nerve to the inferior oblique muscle may be susceptible to injuries on its course along the lateral border of the IR [41]. Thus, surgical manipulations performed on the IR may cause palsy of the inferior oblique muscle and may also trigger tonic pupil due to damage of parasympathetic fibers that most commonly course with fibers destined for the inferior oblique muscle [41]. Moreover, if the nerve to the inferior oblique pierces the IR, its susceptibility to injury associated with IR stretching seems to be greater [41].

**Figure 4:**
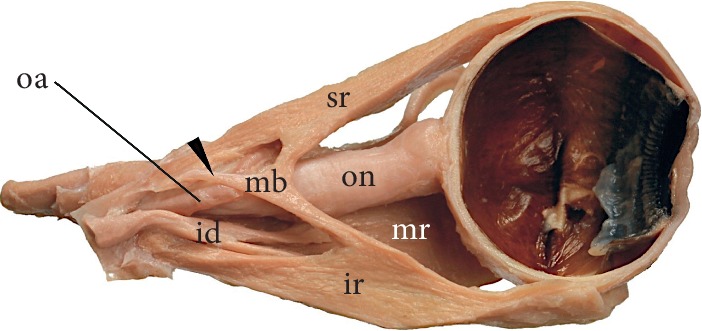
Superior (sr) and inferior (ir) rectus muscles connected to each other by muscular bands (mb). Such muscular bands, taking origin (marked by black arrowhead) from the common tendinous ring and attached to rectus muscles are referred to as accessory rectus muscles. The characteristic feature of accessory rectus muscles is that they are located laterally to the optic nerve (on). Sagittal section of the specimen taken from the right orbit. id: inferior division of the oculomotor nerve; mr: medial rectus muscle. This figure is a modification of the drawing taken from Haładaj et al. [43] under the terms of the Creative Commons Attribution 4.0 International License (https://creativecommons.org/licenses/by/4.0/), which permits unrestricted use, distribution, and reproduction in any medium.

Occasionally some muscular slips linking vertical ERMs may be observed [28, 42–44]. Various types of such atypical muscular bands were reported in the literature. The first group of those anomalies involves cases in which the SR and IR are connected to each other by muscular bands (Figure 4) [28, 42]. In some cases, those muscular slips may also be attached to the annulus of Zinn. Such muscular bands, taking origin from the common tendinous ring and attached to rectus muscles are referred to as accessory rectus muscles [28, 42, 43]. The characteristic feature of accessory rectus muscles is that they are located laterally to the optic nerve. In rare instances, an anomalous muscle bundle from the inferior oblique (referred to as “obliquus accessorius inferioris”) may occasionally join the inferior rectus [42]. Occurrence of those anomalies may be explained by deviated differentiation of the superior and inferior mesenchymal complexes during early stages of development [44].

Clinical significance of accessory muscular bands or bridges between the SR and IR depends on their size and location [45]. Reports on such findings vary regarding absence or presence of ocular movement disorders. One of the known cases includes a case report of bilateral muscular slips between superior and inferior rectus muscles in a 68-year-old male cadaver with no eye movement abnormalities reported in the medical history [43]. Also, Kightlinger et al. [45] found a series of cases of muscular bands linking the SR and IR. Those cases were discovered as incidental findings, with no relation to the clinical symptoms experienced by examined patients [45]. Similar cases of anomalous muscular bands linking the SR and IR with no clinical manifestation were reported by Von Lüdinghausen et al. [46] and Kakizaki et al. [47] (“in a 45-year-old female cadaver, who had no ocular movement disorders in her lifetime”). Kakizaki et al. [47] suggest that muscular bridges linking the SR and IR “are important in the differential diagnosis of intraorbital space-occupying lesions, rather than the differential diagnosis of strabismus.” During diagnostic imaging, such anomalous muscular slips may be confused with blood vessels or various pathologies within the orbit (for example, lymphoma, vascular malformations, or metastasis) [43, 45]. Based on the report of Khitri and Demer [48] the muscular slips connecting the SR with the IR represent about 33% of all types of bands detected within the orbit.

#### 2.3.2. Horizontal Rectus Muscles

Based on descriptions of Bergman et al. [42] and Kocabiyik [28], major anatomical variations of the horizontal rectus muscles involve duplication of the LR, bifid insertion of the MR, as well as the absence of the LR or MR. However, numerous congenital abnormalities of the horizontal rectus muscles were reported in the medical literature. For instance, Sachdev et al. [49] conclude that numerous congenital anomalies (such as absence, hypoplasia, bifurcation, or duplication) may involve ERMs, including the LR. Those authors presented a case of a 5-year-old girl with multiple associated ocular and systemic congenital malformations who had posteriorly malinserted LR at 15 mm from the limbus in the right eye [49]. Park and Oh reported the case of an accessory LR in a patient with congenital third-nerve palsy [50]. Liao and Hwang [51], in turn, described a case of an accessory LR in a 51-year-old woman with normal ocular motor control. In this case, supernumerary LR was unilateral and measured approximately 10% the size of a normal LR. An accessory LR described by Liao and Hwang [51], originated in the apex of the orbit, was located between the optic nerve and the LR and had an insertion to the superolateral aspect of the eyeball. Congenital absence of the LR was reported by Sandall and Morrison [52], while an absence of a lateral rectus muscle associated with duplication of the chromosome segment 7q32----q34 was reported by Keith et al. [53]. A case of an accessory MR in strabismus fixus convergens was described by Lee and Kim [54]. Murthy [55] reported two patients, one with bilateral congenital dystrophia of the MR and one with bilateral absence of the MR; In addition, in one of those cases absence of the LR was observed.

According to Bergman et al. [42], an additional muscular slip may also occur between the LR and MR. Such muscular connection usually passes across the posterior third of the orbit, beneath the optic nerve, and fuses with the MR. Detailed anatomy of the ligamentous bands between the LR and SR, in turn, was presented by Nam et al. [56]. While muscular bands between the SR and IR do not have to cause eye movement disorders (as described in the previous paragraph), Lueder [57] states that anomalous orbital structures (such as muscular or fibrous bands) that attach to the globe may produce a mechanical restriction, resulting in incomitant motility disorders. In rare instances, those anomalous structures may also cause a strabismus [57]. Generally, three types of anomalous orbital bands may be distinguished [57]. Fibrous or muscular bands may be located beneath the ERMs; they also may originate from the ERMs themselves and insert in abnormal locations or may occur as discrete anomalous muscles that take origin in the posterior orbit and insert in abnormal locations on the globe. According to Lueder [57] orbital bands may be responsible for unusual patterns of strabismus, which is confirmed by clinical findings. Anomalous orbital structures may be found in cases of globe retraction not associated with Duane retraction syndrome, very severe vertical strabismus, or an elevated deficit deepening in the abduction. In the study of Khitri and Demer [48], unilateral or bilateral orbital bands were found in 0.8% orthotropic and 2.4% strabismic patients. Based on the findings of Khitri and Demer [48], it may be concluded that horizontal bands linking the MR with LR immediately posterior to the globe may limit supraduction by collision with the optic nerve and, which is particularly important, those bands are usually located too deep to be approached via conventional strabismus surgical approaches.

### 2.4. Vascular Supply of ERMs

**Figure 5:**
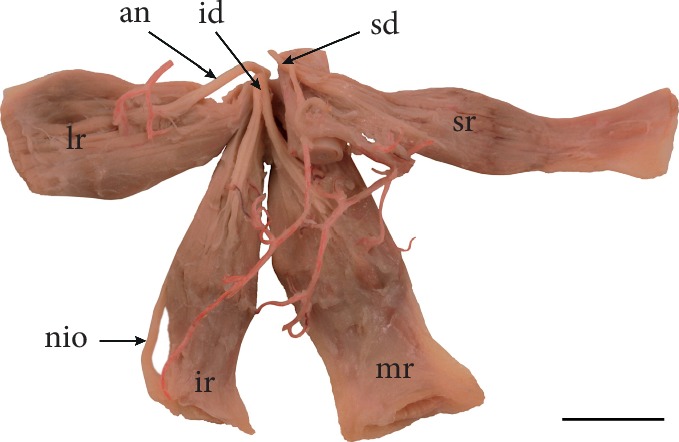
General arrangement of vascularization and innervation of extraocular rectus muscles. Arteries have been injected by red resin to expose muscular branches reaching individual muscles. Specimen taken from the right orbit. Scale bar shows 10 mm. an: abducens nerve; id: inferior division of the oculomotor nerve; ir: inferior rectus muscle; lr: lateral rectus muscle; mr: medial rectus muscle; nio: nerve to the inferior oblique muscle; sd: superior division of the oculomotor nerve; sr: superior rectus muscle.

According to the description provided by Shumway et al. [11], the ERMs are vascularized by the blood vessels that also supply nearly all lateral half of the anterior eye segment. However, the medial half of the anterior eye segment also shares vascular supply with ERMs [11]. ERMs are supplied mainly by the muscular branches taking origin from the ophthalmic artery (Figure 5) [7–11, 58, 59]. According to Fernández Cabrera and Suárez-Quintanilla [8] the LR may receive additional blood supply directly from the lacrimal artery, which is a branch of the ophthalmic artery. Based on arterial vascularization of the extraocular muscles provided by Erdogmus and Govsa [59], the most common origins of muscular branches are the lacrimal artery for the inferolateral muscular trunk (43.36%), the bend of the ophthalmic artery for the superior oblique (36.84%), the supraorbital artery (36.84%) for levator palpebrae superioris, the distal end of the ophthalmic artery for the SR (52.6%), the lacrimal artery for the LR (89.47%), and the inferomedial muscular trunk for the MR (84.51%). Venous drainage of ERMs is provided by the superior and inferior orbital veins [8–11, 58]. Current reports suggest “laminar structure” of ERMs, wherein the orbital and global layers of ERMs are believed to serve different functions [60]. Oh et al. [60] proved that the orbital layer within ERMs had “significantly more vessels per unit area, more vessels per fiber, and more total vascular luminal area, than the global layer.” Based on those findings, Oh et al. [61] suggest that higher blood flow in the orbital layers may correlate with the greater metabolic activity of this area.

### 2.5. Specific Innervation Pattern of ERMs

#### 2.5.1. General Arrangement of ERMs Nerves

The microsurgical anatomy of the ocular motor nerves is relevant in the clinical context, helping to plan surgical accesses that save ERMs function or helping to better understand the symptoms of nerve palsy. The somatomotor innervation of ERMs comes from two different sources. Three out of four ERMs are innervated by the third cranial (oculomotor) nerve. Innervation of the SR is provided by the superior division (branch) of the oculomotor nerve, while muscular branches to the MR and IR are derived from the inferior division (branch) of the oculomotor nerve. The LR, in turn, is innervated by the sixth cranial (abducens) nerve [7–11].

The two main branches (divisions) of the oculomotor nerve join the orbit through the superior orbital fissure, medially to the lacrimal nerve. The superior division of the oculomotor nerve enters the inferior surface of the SR with 5 (from 3 to 7) muscular subbranches which continue their course within the muscle belly (Figure 5) [60, 62]. Some of those fibers continue their course around the medial border of the SR (or pierce the muscle) to innervate the overlying levator palpebrae superioris muscle [39, 40]. The inferior division of the oculomotor nerve gives two muscular branches to the ERMs, nerve to the inferior oblique muscle and, occasionally, the parasympathetic root of the ciliary ganglion. One of those muscular branches passes forward, to the IR. Typically, about seven subbranches (range 3–10) reach the internal surface of the IR [60, 62]. The second muscular branch originating from the inferior division of the oculomotor nerve passes medially, below the optic nerve, and reaches the MR. According to Zhang et al. [60] the muscular branch to the MR may be divided into five subbranches (from 3 to 8) reaching the internal surface of the muscle. The abducens nerve innervates only the LR. It enters the orbit via the superior orbital fissure, below the nasociliary nerve.

#### 2.5.2. New Anatomical Data on ERMs Intramuscular Innervation Pattern

The latest anatomical works on innervation of ERMs draws special attention to the intramuscular distribution of the somatomotor nerve fibers, which is of importance for understanding the complex action of those muscles. Innervation of ERMs appears to have some special properties, such as high ratio of nervous branches (1 to 3 or 1 to 5, compared to other skeletal muscles which are 1 to 50 or even 1 to 125) [10, 11, 63] or potential independent activity of muscular layers and compartments [2, 6, 18–22, 64, 65]. The latest property was widely discussed regarding the intramuscular innervation of the LR.

**Figure 6:**
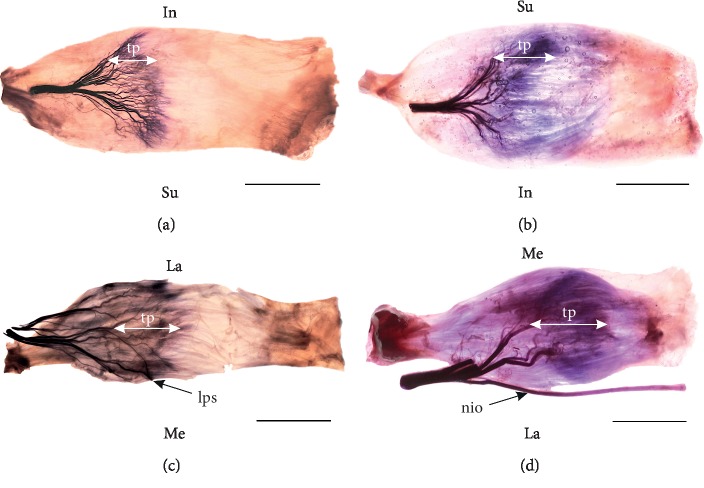
The course and arrangements of the intramuscular nerves within the extraocular rectus muscles based on Sihler's stain. The terminal plexus (tp) has been marked. Scale bar shows 10 mm. (a) Specimen of the right lateral rectus muscle; (b) specimen of the right medial rectus muscle; (c) specimen of the right superior rectus muscle; (d) specimen of the right inferior rectus muscle; lps: muscular branches to the levator palpebrae superioris muscle wrapping around the medial border of the superior rectus muscle; nio: nerve to the inferior oblique muscle travelling along the lateral border of the inferior rectus muscle; In: inferior direction; La: lateral direction; Me: medial direction; Su: superior direction.

Detailed anatomy of the abducens nerve (including intramuscular distribution of nervous branches) in the LR was examined in detail by Nam et al. [3, 4]. According to those authors, the abducens nerve joins the LR on posterior one-third of its length and then divides into a few muscular subbranches, while the intramuscular nerve course finishes around the half of the LR length [4]. Similar results on intramuscular nerve distribution in the MR were obtained in the only (to date) study carried out by Shin et al. [5]. Based on this report it may be concluded that muscular subbranches of the oculomotor nerve join the MR at a mean of two-fifths of its length and then typically divides into a few intramuscular divisions. Despite the detailed analysis of the literature, no reports were found on the course of intramuscular nerves within SR and IR on human material. However, the analysis of specimens stained by Sihler's method prepared especially for this review suggests that, apart from minor differences, all four ERMs show similar general pattern of intramuscular distribution of nerves subbranches (Figure 6). Based on previous descriptions provided by Shin et al. it may be stated that within the ERMs “The intramuscular nerve distribution showed a *Y*-shaped ramification with root-like arborization” (Figure 6). After the muscular subbranches enter the muscle belly, they undergo numerous further divisions forming the dense area of intramuscular nerves showing plexiform appearance [3–5]. This conglomerate of intramuscular nerves forms so-called “terminal plexus” which ends at about half the length of the muscle (Figure 6). In two studies conducted on the LR, the presence of single thin nervous branches joining the areas of LR's attachments was suggested. Some studies [2–4, 6, 64] suggest also some specific segregation of the abducens nerve's muscular subbranches into the superior and inferior groups. Those findings may provide a good understanding of the intramuscular anatomy and action of the ERMs.

## 3. Summary

The latest research complements the classic anatomical descriptions of ERMs, shedding new light on the complex structure and function of those muscles. Precise control of the eyeball movements is based on a complex anatomical and biomechanical system which involves specific structure, innervation, and vascularization, as well as diverse activity of the ERMs.
